# Theoretical and electrochemical evaluation of tetra-cationic surfactant as corrosion inhibitor for carbon steel in 1 M HCl

**DOI:** 10.1038/s41598-023-27513-7

**Published:** 2023-01-18

**Authors:** A. Elaraby, Shrouk. Abd El-samad, Eman. A. khamis, E. G. Zaki

**Affiliations:** 1 Egyptian Petroleum Research Institute, Nasr City 11727, Cairo, Egypt; 2 University College of Umluj, University of Tabuk, Tabuk, Saudi Arabia

**Keywords:** Environmental chemistry, Corrosion

## Abstract

Recently, scientist study the role of surfactants for carbon steel corrosion protection. In the present study, newly tetra-cationic surfactant (*CS4*: 1,N1'-(ethane–1,2-diyl) bis (N1, N2—didodecyl–N2–(2- (((E)-3-hydroxy-4-methoxy-benzylidene)amino)ethyl)ethane-1,2-diaminium) chloride) based on Schiff-base compound(5,5'-((1E,17E)-2,5,8,11,14,17-hexaazaoctadeca-1,17-diene-1,18-diyl)bis(2-methoxyphenol) was synthesised, purified and characterized using FTIR and ^1^HNMR spectroscopy. The synthesized Tetra-cationic surfactant (*CS4*) was evaluated as anti-corrosion for carbon steel (*CS*-metal) in aggressive 1 M HCl using electrochemical impedance spectroscopy (EIS) and potentiodynamic polarization techniques (PDP). *CS4* compound had a good surface-active property by reducing the surface tension as a result to the hydrophobic chains role. The prepared *CS4* behaved as hybrid inhibitor (mixed-type) by blocking the anodic and cathodic sites. *CS4* exhibited good inhibition efficiency reached 95.69%. The surface morphology of *CS*-metal was studied using scanning electron microscopy (SEM) and X-ray photoelectron spectroscopy (XPS)confirming the anti-corrosive effect of *CS4* compound returned into the adsorption process of *CS4* molecules over *CS*-metal which obeyed Langmuir adsorption isotherm. The inhibitive effect of *CS4* was supported by theoretical quantum chemical studies using the density functional theory (DFT), Monte Carlo (MC) and Molecular Dynamic (MD) simulation.

## Introduction

Carbon steel (*CS*-metal) is the most important portion of engineering alloys and steel production besides its application, it is widely used in different areas of industry such as petroleum industry^1^. Carbon steel corrosion process in acidic surroundings is a big issue in petrochemical, marine or chemical production because of the use of acidic solution of hydrochloric acid (HCl) for oil well and pipelines cleaning^2^. So, carbon steel protection is a desired process. One of the most attractive option that can be applied is using of Surfactants that are characterized by their unique chemical complex which qualifies it to have a great importance in many fields, whether industry or research^3,4^. Surfactants (surface active agents) are organic compounds with two various parts with different polarities attached together, one of them is high polar (hydrophilic head) while the other featured by its hydrophobicity (hydrophobic tail)^5,6^. Surfactants are known with their ability to reduce the effect of surface tension on surface between two phases besides their ability to either gathered in micelle forms and adsorb in the interfaces. The unique structures of surfactant with numerous properties play an important role in many of different applications such as pharmaceutical industry, detergent, petroleum oil recovery, demulsifier and corrosion inhibitors^7^. One of these types of surfactants is cationic surfactant where the hydrophilic part bears appositive charge. This type is characterized by its ability to use as corrosion inhibitor in acidic medium^8,9^.

Corrosion is as a destructive attack of metals due to interaction with environment during industrial process^10,11^. Metals in contact with corrosive environment suffer from short life extent because of corrosion process occurs^12^.

Corrosion inhibition is an important strategy for long-term protection of metals. In order to reduce the corrosion process of metals, several techniques have been applied. Corrosion inhibitors are one of the simplest and most efficient approaches to protect *CS*-metal during treatment process (cleaning, pickling and descaling). A large number of molecules have been tested for corrosion inhibition in past decades. According to the numerous advantages of using corrosion inhibitors for *CS*-metal protection from corrosive surrounding. Therefore, it has received extensive attention from corrosion protection scientists^13^. Corrosion inhibitors are materials added to corrosive environment in small concentrations, decrease the corrosion rate of metals by decreasing the reaction of the metal with the environment via formation of a protective adsorbed layer^13,14^. Scientists concern is to design an appropriate highly efficiencies, low cost and eco-friendliness corrosion inhibitor, fundamentally depends on the chemical structure of the surfactant which contain electronic rich functional groups like hetero atoms (N, O, S or P), double bond and aromatic rings as an active centres during the adsorption process^1,2,7^. Surfactants based on Schiff-base containing C = N^15–17^ have many of the above characteristics paired with a structure which make them promising effective corrosion inhibitors^18,19^.

Hegazy et al.^17^ studied the anti-corrosion of three new synthesized cationic surfactants (I(4 N), II(4 N) and IV(4 N)) for carbon steel using weight loss, electrochemical impedance spectroscopy (EIS) and polarization measurements in 1 M HCl. The prepared compounds exhibit high protection against corrosive HCl. EIS result show that the inhibition efficiency of IV(4 N) compound reached to about 96%. El-Dougdoug et al.^20^ discussed the inhibition performance of mono, di and tetra cationic surfactants for carbon steel in 1 M HCl. The inhibition efficiency reached to about 96 and 91% using mono and tetra cationic surfactants respectively. The inhibition performance of the prepared compounds was studied through harsh conditions (different temperatures and immersion time) showed high protection for carbon steel due to protective film layer of the adsorbed inhibitors. Ting Zhou et al^21^. discussed the inhibitory behaviour of imidazolium Gemini surfactant on carbon steel in HCl solution using electrochemical EIS, polarization curves, weight loss measurement and the quantum chemical study. Results reveal that the inhibition efficiency of the inhibitor reaches 96% and the quantum chemical calculation was employed to interpret the possible inhibition mechanism of the prepared inhibitor.

The novelty of our study is the synthesis of tetra-cationic surfactants (*CS4*) enriched with variable function groups N = C, NH, OH, O–CH_3_ and aromatic ring through two simple steps. The prepared compound was evaluated as corrosion inhibitor for *CS*-metal in 1 M HCl solution using EIS (electrochemical impedance spectroscopy) and PDP (potentiodynamic polarization). The surface analysis of *CS*-metal was studied using scanning electron microscope (SEM) and X-ray photoelectron spectroscopy (XPS) which confirmed the inhibition performance of the prepared *CS4* in 1 M HCl. The computational investigation of *CS4* as corrosion inhibitor was studied using the density functional theory (DFT), Monte Carlo (MC) and Molecular Dynamic (MD) Simulation in gas and liquid phases.

## Materials and experimental techniques

### Materials

All chemicals were used without further purification, 3-hydroxy-4-methoxybenzaldehyde, pentaethylenehexamine, and chlorododecane were purchased from Sigma Aldrich Company.

### Synthesis of cationic surfactant

**Scheme 1:**
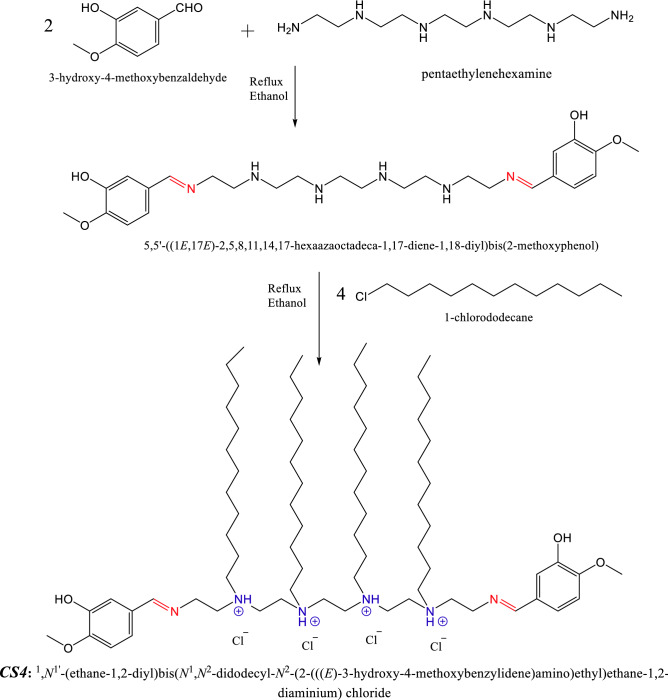
Synthesis of *CS4*.

Tetra-Cationic surfactant (*CS4*) was prepared as in Scheme 1 through two steps. First step: 0.02 M 3-hydroxy-4-methoxybenzaldehyde was refluxed with 0.01 M pentaethylenehexamine in presence of ethanol as a solvent for 8 h. The solvent was evaporated then washed using petroleum ether to obtain a reddish-brown semisolid compound (Schiff-base)^22^.

Second step: The obtained Schiff base was refluxed with 1-chlorododecane in ratio 1:4 and ethanol as a solvent for 48 h to produce the corresponding cationic surfactant (*CS4*). The obtained *CS4* compound was purified using diethyl ether and n-hexane^17^. The chemical structure was confirmed by FTIR and ^1^HNMR.

### Surface active parameters

Surface tension (γ) for different concentrations of *CS4* compound was measured in 1 M HCl solution at room temperature using Theta instrument. Surface active parameters such as π_**CMC**_ (The effectiveness), Γ_max_ (The maximum surface excess), A_min_ (minimum surface area), CMC (critical micelle concentration), and the change in free energy of micellization ${(\Delta G}_{\mathrm{mic}}^{^\circ })$ and adsorption ${(\Delta G}_{\mathrm{ads}}^{^\circ })$ were calculated and discussed.

### Electrochemical measurements

Mild carbon steel specimens with chemical composition in wt.%; C = (0.12∼0.20), Si = (0.20∼0.55), Mn = (1.20∼1.60), P = (≤ 0.045), S = (≤ 0.045), Cr = (≤ 0.30), Ni = (≤ 0.30), Cu = (≤ 0.30) and Fe balanced with a surface pre-treatment procedure was carried out prior to each experiment, The corrosion behavior of CS-metal was studied in 1 M HCl solution with and without different concentrations (1 ppm: 50 ppm) of *CS4* compound using OrigaMaster 5 potentiostat/galvanostat. Three electrode system of *CS*-metal as working electrode, Pt (platinum) electrode as auxiliary electrode and Ag/Agcl as a reference electrode were connected to origalys instrument. After OCP (30 min), EIS (electrochemical impedance spectroscopy) and PDP (potentiodynamic polarization) were performed. EIS was measured in frequency range (100 kHz and 0.05 Hz) and PDP was measured using potential range ± 300 mV around OCP value at 2 mV/s.

### Computational studies

In the computational investigation, Accelrys, Inc.’s BIOVIA Materials Studio (7.0) program was employed. The optimization geometry of *CS4* was carried out in two different phases (gas and solution) using the DMol3 module with Perdew and Wang (LDA) exchange–correlation functional and DND-3.5 basis set. The interaction between *CS4* and iron surface (1 1 0) with 30 Å vacuum layer was achieved and simulated in a corrosion environment^23^. Some quantum chemical parameters of *CS4* such as: the electron density, HOMO (the highest occupied molecular orbital), LUMO (the lowest unoccupied molecular orbital) and dipole moment which are used to predict the most reactive centres in *CS4* compound were calculated.

### SEM

The *CS*-metal surface morphology was examined for more information about the corrosion process and the inhibition performance of *CS4* using SEM (Scanning Electron Microscope, ZEISS) as a powerful tool to confirm the performance of *CS4* as an efficient inhibitor. The *CS*-metal samples were scanned after immersion in 1 M HCl solution with and without 50 ppm of *CS4* at room temperature for 6 h.

### XPS

XPS is a surface quantitative spectroscopic technique for interface of metal/solution analysis. *CS*-metal samples with exposed surface area 1 cm^2^ were immersed in 1 M HCl solution for 24 h in the absence and presence of CS*4* inhibitor and analysed using a KRATOS XSAM-800 to determine the bonding characteristics of CS*4* molecules on the metal surface.

## Result and discussion

### Structure characterization

#### FTIR

**Figure 1:**
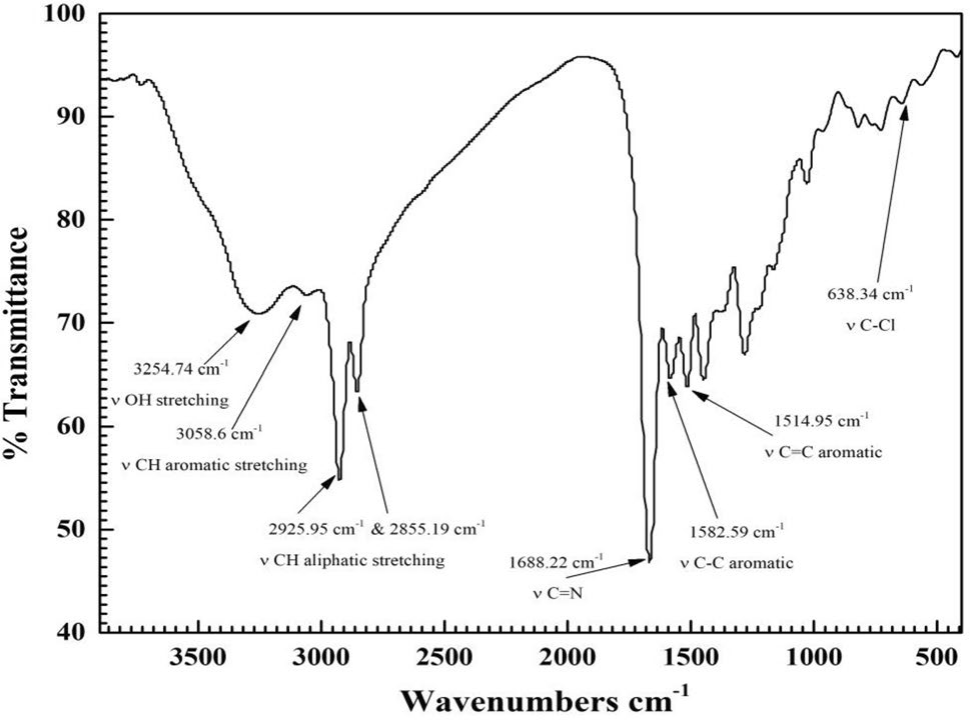
FTIR spectrum of *CS4* compound.

*CS4* chemical structure was confirmed by Fourier transform infrared spectroscopy as in Fig. 1 showed bands at 3254.74 cm^−1^ (ν OH-stretching), 3058.6 cm^−1^ (νC-H aromatic stretching), 2925.9 cm^−1^ and 2855.19 cm^−1^ (νC–H aliphatic stretching), 1668.22 cm^−1^ (νC = N), 1582.59 cm^−1^ (ν C–C aromatic), 1514.95 cm^−1^ (νC = C aromatic), 1448.9 cm^−1^ (νCH_2_), 1029.12 cm^−1^ (νC-O), 638.34 cm^−1^ (νC–Cl).

#### ^1^HNMR

**Figure 2:**
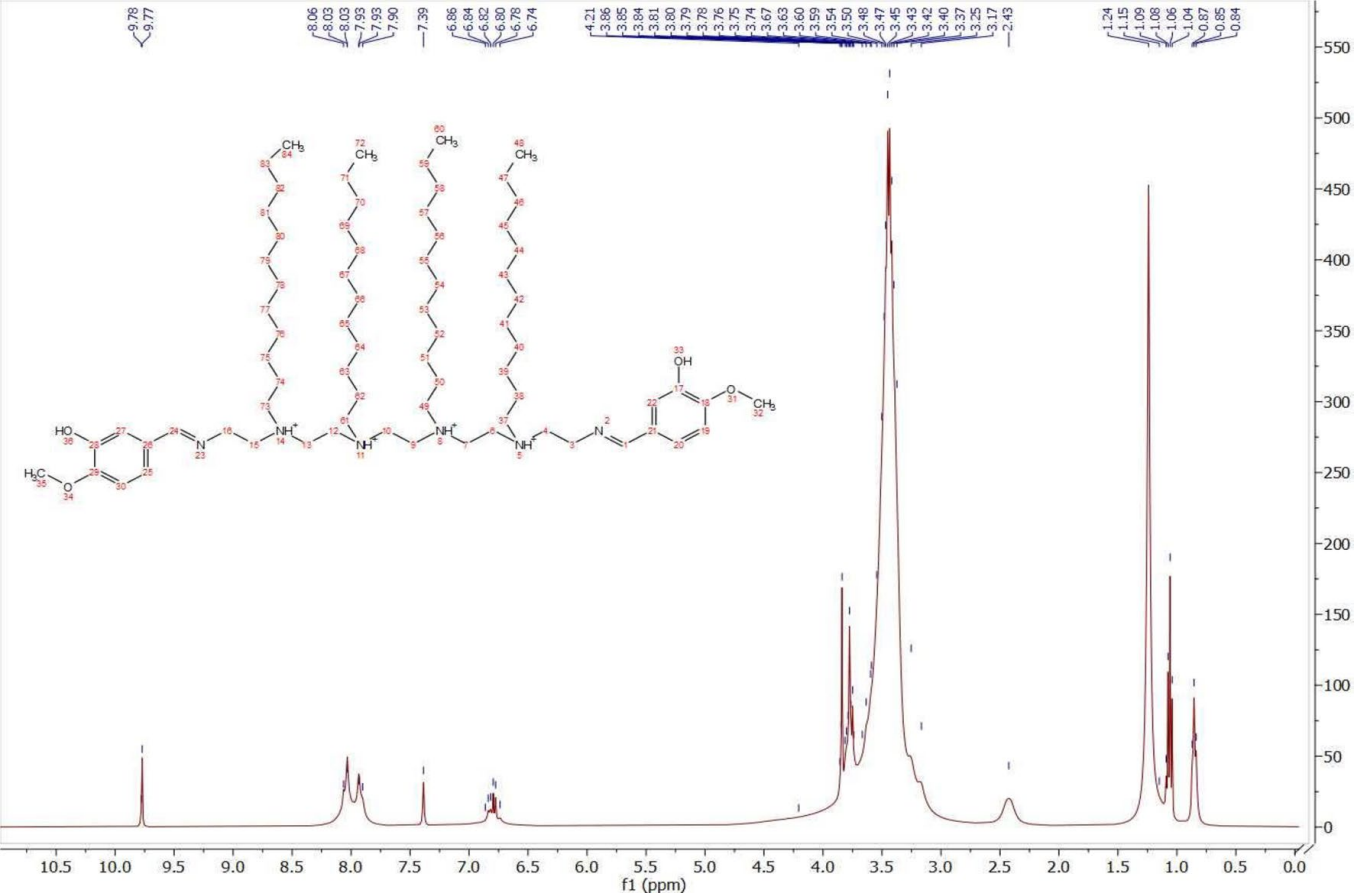
^1^HNMR spectrum of *CS4* compound.

^1^HNMR spectra of *CS4* was characterized by Bruker High Performance Digital as in Fig. 2 (δ, ppm): 0.84 (m, 12H, (CH_2_)_n_C**H**_3_), 1.06 (m, 36H, (C**H**_2_)_9_CH_3_), 1.24 (m, 8H, **CH**_**2**_(CH_2_)_9_CH_3_), 3.4 (m, 8H, ^+^NH**CH**_**2**_CH_2_(CH_2_)_9_CH_3_), 3.75 (t, 6H, O**CH**_**3**_), 3.86 (m, 4H, **CH**_**2**_N = C), 6.86–7.93 (d, 6H, **Ar**), 7.39 (s, 4H,^+^N**H**), 8.06 (s, 2H, N = **CH**), 9.78 (s, 2H, **OH**).

### Surface active parameters

**Figure 3:**
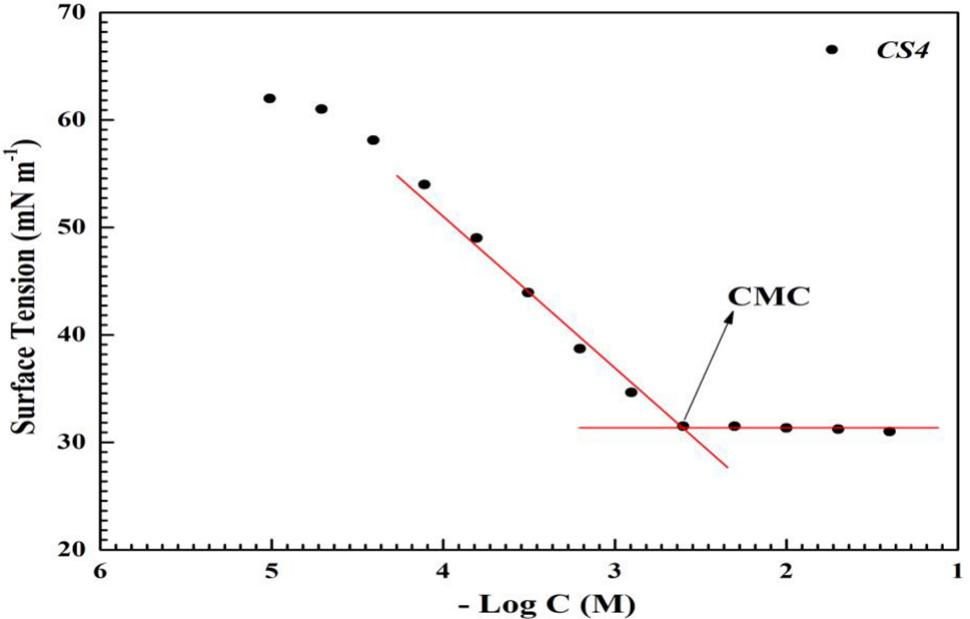
Surface tension Vs concentration of *CS4* in 1 M HCl at room temperature.

Surface tension values (γ) of the prepared *CS4* compound was measured at room temperature (298 K) as in Fig. 3 showing the relationship between Surface tension (γ) and various concentrations of *CS4* (-log C). The surface tension (${\gamma }_{\mathrm{o}}$) decreased with addition of various concentrations till *C*_CMC_ as seen in Fig. 3 (γ = 31.5 at *C*_CMC_ = 2.5 × 10^−3^ M) while no change was observed after *C*_CMC_. This decrease was due to the migration of *CS4* molecules to the solution interface because of presence of four hydrophobic fatty chain in its structure^24^. CMC was determined by the intersection between two lines as in Fig. 3 (before and after *C*_CMC_)^25^. Values of π_**CMC**_, Γ_max_, A_min_, ${\Delta G}_{mic}^{o}$ and ${\Delta G}_{\mathrm{ads}}^{^\circ }$ were calculated and listed in.

Table 1 as the following equation:

1 $${\pi }_{\mathrm{CMC}}={\upgamma }_{\mathrm{o}}- {\upgamma }_{\mathrm{CMC}}$$

2 $${\Gamma }_{max}=(-\frac{d{\varvec{\gamma}}}{\mathit{dln}C})/\left(2.303nRT\right)$$

3 $${A}_{min}={10}^{14}/{{N}_{\mathrm{A}}\Gamma }_{\mathrm{max}}$$

4 $${\Delta G}_{mic}^{o}=2.303RT \,\mathrm{log} \, CMC$$

5 $${\Delta G}_{\mathrm{ads}}^{^\circ }={\Delta G}_{mic}^{^\circ }-(0.06023 \times {\pi }_{\mathrm{CMC}}/{\Gamma }_{max})$$

where γ_CMC_, n, *R*, *T* and *N*_A_ are surface tension of CS4 at CMC, the slope of the straight line (*γ* Vs –log *C*), number of ions dissociation (n = 5), the gas constant, the absolute temperature (K) and Avogadro’s number^26^. The value of ${\pi }_{\mathrm{CMC}}$ in Table 1 showed that *CS4* compound decreased surface tension effectively due to presence of large hydrophobic part in its structure^27^. the values of ${\Gamma }_{max}$ and ${A}_{min}$ indicated that *CS4* molecules occupied large surface area at interface as result in the role hydrophobic carbon chain which mean that *CS4* compound has adsorption affinity at interface^28^. The -ve values of ${\Delta G}_{mic}^{^\circ }$ and ${\Delta G}_{\mathrm{ads}}^{^\circ }$ as in Table 1 indicated that spontaneous process of micellization and adsorption occurred. Also, ${\Delta G}_{\mathrm{ads}}^{^\circ }$ value was more than that of ${\Delta G}_{mic}^{^\circ }$ indicating that the prepared *CS4* prefer adsorption than micellization confirming that *CS4* can act as corrosion inhibitor^16,26^.

**Table 1:** surface active parameters of *CS4* in 1 M HCl at room temperature.

*Inh*	*γ*_cmc_ (mN m^−1^)	*π*_cmc_ (mN m^−1^)	*Γ*_max_ × 10^−3^ (mol cm^-2^)	*A*_min_ × 10^−7^ (nm^2^)	*C*_cmc_ × 10^−3^ (Mol/L)	${\Delta G}_{\mathrm{mic}}^{\mathrm{o}}$ kJ.mol^−1^	${\Delta G}_{\mathrm{ads}}^{\mathrm{o}}$ kJ.mol^−1^
*CS4*	31.5	41.2	1.508	1.102	2.5	− 14.595	− 16.243

### Electrochemical corrosion tests

#### Electrochemical impedance spectroscopy

**Figure 4:**
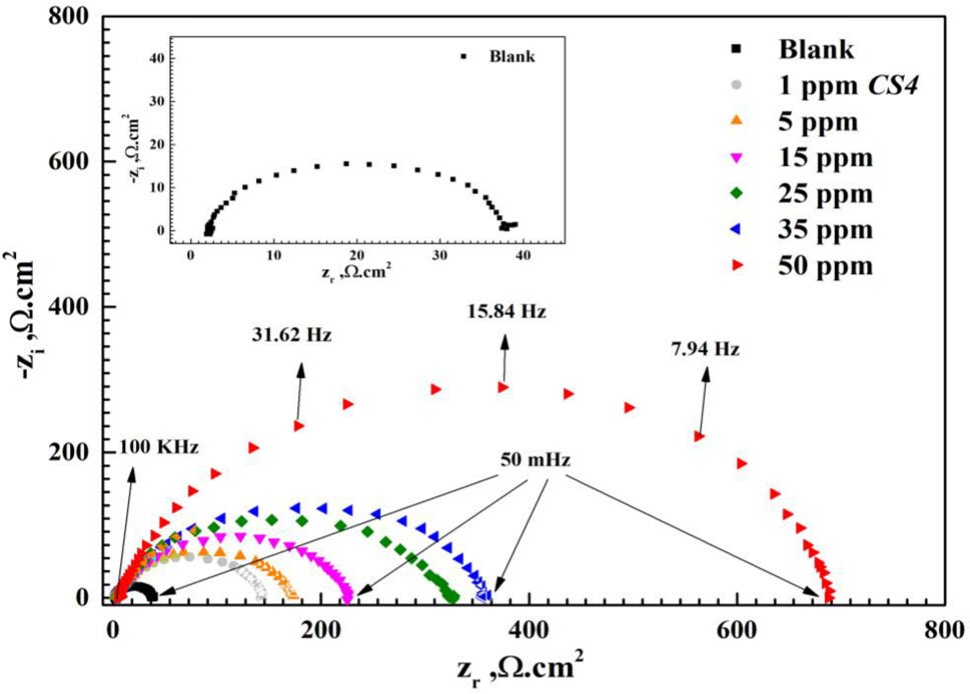
Nyquist plots for *CS*-metal in 1 M HCl in the absence and presence of various concentrations of *CS4* at room temperature.

**Figure 5:**
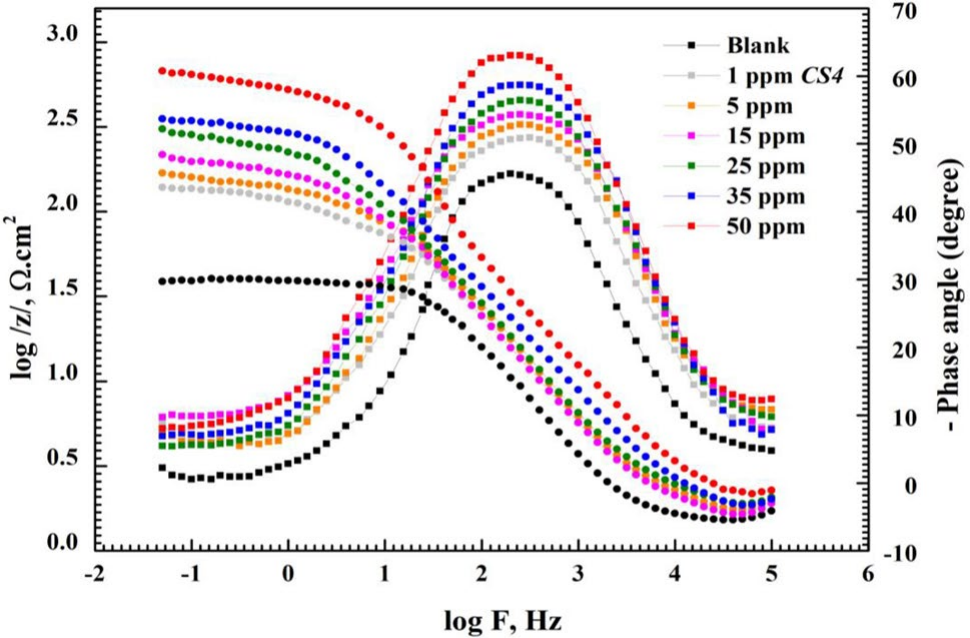
Bode and phase diagram for *CS*-metal in 1 M HCl in the absence and presence of various concentrations of *CS4.*

Nyquist and bode diagrams as in Figs. 4 and 5 exhibit corrosion behaviour of *CS*-metal in destructive free acidic HCl solution and with *CS4* inhibitor at room temperature. It can see that the diameter of semicircle increases as concentration of *CS4* compound increase which can be explained as number of adsorbed molecules on *CS*-metal surface increase and more surface coverage of *CS4* on *CS*-metal through adsorption process which verifies that corrosion behaviour is controlled and affected by *R*_ct_ (charge transfer resistance)^16,29^. No change in Nyquist diagram in absence and presence of *CS4* indicating that the corrosion mechanism of *CS*-metal not affected by the addition of *CS4* compound and controlled by *R*_ct_^30,31^. The increase in capacitive loop diameter confirms the higher inhibition/protection of *CS*-metal and subsequently decrease in corrosion rate in acidic HCl solution in presence *CS4* compound^32^. Furthermore, the compressed semicircle shape of Nyquist diagram as in Fig. 4 may be a result of surface roughness and in-homogeneities of *CS*-metal^33^.

**Figure 6:**
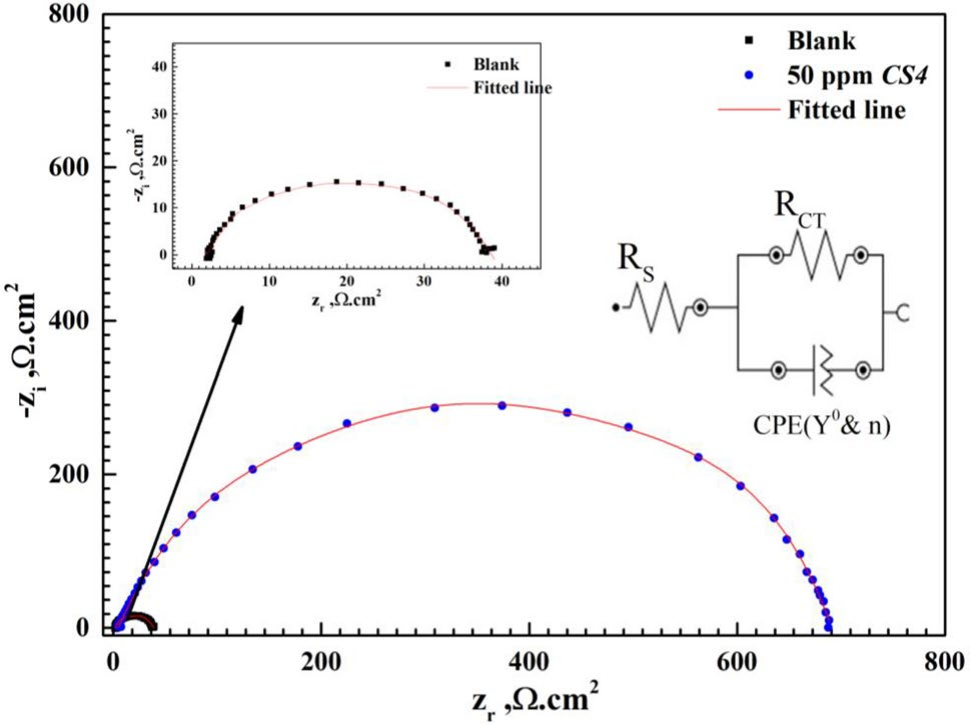
Nyquist plots of *CS*-steel in 1 M HCl with and without high concentration (50 ppm) of *CS4* using the proposed equivalent circuit.

At low frequency region, *R*_ct_ value increase after the addition of *CS4* concentrations compared to the blank solution. Also, the noticed change in bode-phase curve can be attributed to the relaxation effect resulting by the adsorption of *CS4* molecules^34^. As seen in Fig. 5 the gap between bode modulus at lower frequency increase with rising of concentration compared to that in free HCl solution. This demonstrates adsorption of *CS4* and protection of *CS*-metal by formation of protective layer against aggressive HCl^33^. While at higher frequency, reduction in corrosion rate of *CS*-metal and high protection efficiency are observed as shift of phase angle to wards -90 with rising of concentration^35,36^. The proposed equivalent circuit was presented as in Fig. 6 using *R*_s_, *R*_ct_ and *CPE* which were extracted and listed in Table 2 indicating that one constant phase element clarified by *Y*_o_ and *n*. In general, for $n=0 \&-1$, Z_CPE_ represents resistance $(R={Y}_{o}^{-1})$ and inductive $(L={Y}_{o}^{-1})$ respectively. while for $n=0.5 \& 1$, Warburg impedance (W = *Y*_o_) and capacitance with (C = Y_o_) for $n=1$^17^,34,35]. From Table 2, the value of *n* decreased by the addition of *CS4* indicated that the surface heterogeneity increases due *CS4* adsorption at the *CS*-steel/solution interfaces. Also, *Y*_o_ decreased with the addition of *CS4* due to increase thickness of adsorbed layer on *CS*-metal^15,16^. The following equation was used to determine *CPE* impedance (*Z*_CPE_):

6 $${Z}_{CPE}={Q}^{-1}(i{\upomega }_{max}{)}^{-n}$$

where *Q* = constant phase element constant, *ω*_max_ = the angular frequency^39^. From Table 2, *R*_S_ values increase after the addition of *CS4* more than that of blank solution indicating that, the solution conductivity decreases with the addition of the studied *CS4*. This denotes that, shielding of *CS*-metal from corrosive solution and greater blocking of the active sites at CS-metal surface by *CS4* compound^40,41^. *R*_ct_ value increase and reached 138.3 Ω.cm^2^ and 681.4 Ω.cm^2^ in presence of 1 ppm and 50 ppm of *CS4* compound respectively, compared to *R*_ct_ of free acid solution 38.605 Ω.cm^2^ indicating that strongly shield of *CS*-metal by adsorbed *CS4* molecules from destructive action of HCl species and formation of barrier layer between *CS*-metal and corrosive media^15,29^. The surface coverage (θ) and the inhibition efficiency (η) values were calculated based on *R*_ct_ values according to equation:

**Table 2:** EIS parameters and inhibition efficiency values for *CS*-metal in 1 M HCl with and without various concentrations of *CS4* compound at room temperature.

*Inh*	*Conc*	*R*_s_, (Ω.cm^2^)	*R*_ct_, (Ω.cm^2^)	*CPE*		θ	$\upeta$
				Y^O^, sn *Ω*^*-1*^* cm*^*−2*^	n		
Blank	00.00	1.763	38.605	478.62	0.6671	–	–
*CS4*	1 ppm	3.202	138.30	421.57	0.4565	0.720	72.08
	5 ppm	3.051	168.06	368.21	0.5509	0.770	77.02
	15 ppm	3.735	222.64	329.86	0.4645	0.826	82.66
	25 ppm	3.74	318.61	189.77	0.6105	0.878	87.88
	35 ppm	2.518	352.06	136.52	0.6597	0.890	89.03
	50 ppm	8.377	681.40	82.894	0.5678	0.943	94.33

7 $$\uptheta =({R}_{ct.inh}-{R}_{ct.blank})/{R}_{ct.inh})$$

8 $$\upeta =\uptheta \times 100$$

The values of η increase with concentration due to adsorption of *CS4* on *CS*-metal till reach 72.08 and 94.33% in presence of 1 ppm and 50 ppm of *CS4* compound respectively confirming that *CS4* retard the corrosion process of *CS*-metal effectively^42,43^. On the other hand, Corrosion mitigation and inhibition performance increase with concentration due to surface coverage of *CS*-metal with *CS4* molecules by blocking active centre of *CS*-metal^44–46^.

Replacement of water molecules by *CS4* over *CS*-metal increase with concentration which lead to increase thickness (*T*) of adsorbed layer of *CS4* according to Helmholtz Equation^44–46^.

9 $${C}_{\mathrm{dl}}=\left(\frac{{\varepsilon }^{^\circ }\varepsilon }{T}\right)A$$

where *A,* ε and ε◦ are electrode surface area, the permittivity of local and air dielectric constant of the electric double layer, respectively^16,34^. The ε value of *CS4* is smaller than that of H_2_O, while the volume is obviously larger than that of H_2_O. So, the water molecules over *CS*-metal were replaced with the adsorbed molecules of *CS4* subsequently decrease of ${C}_{\mathrm{dl}}$^34^.

#### Potentiodynamic polarization (PDP)

**Figure 7:**
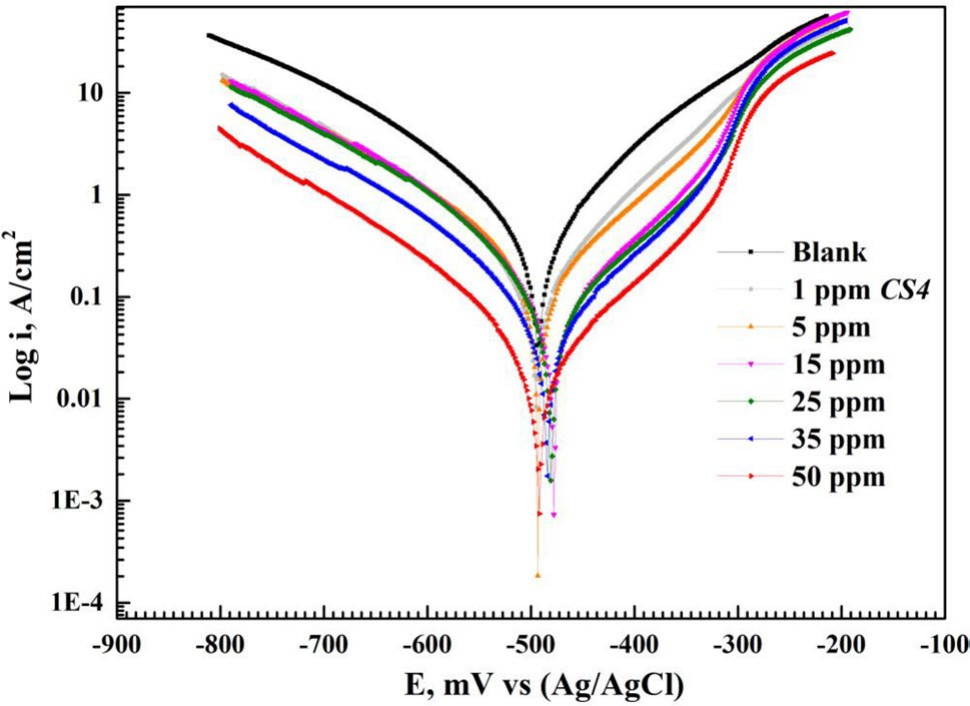
I-V curves for *CS*-metal in 1 M HCl with and without various concentrations of *CS4* at room temperature.

I-V curves as in Fig. 7 sowed the inhibition performance of *CS4* compound for *CS*-metal after fixed immersion time in 1 M HCl. Some electrochemical parameters were extracted from Fig. 7 such as *E*_corr_ (corrosion potential), *β*_c_ (cathodic slope), *β*_a_ (anodic slope), i_corr_ (corrosion current density), and η (inhibition efficiency) listed in Table 3 The values of η were calculated from equation:

10 $$\theta =({\mathrm{i}}_{\mathrm{corr}.\mathrm{blank}}-{\mathrm{i}}_{\mathrm{corr}.\mathrm{inh}})/{\mathrm{i}}_{\mathrm{corr}.\mathrm{blank}},$$

11 $$\upeta =\theta \times 100,$$

where i_corr.blank_ and i_corr.inh_ are the corrosion current densities of free acid solution and *CS4* respectively^47^. Cathodic reaction (H_2_ evolution) and anodic reaction were reduced after *CS4* addition to acidic media confirming the inhibition effect of *CS4*^16^. I-V curves were shifted to lower current values with rising concentration. Parallel line of cathodic Tafel lines confirming that corrosion mechanism not affected by addition of *CS4* inhibitor while the anodic Tafel line reflected the inhibition potency of *CS4* compound^48,49^. It was noticed that at higher anodic potential more than -0.285 V the desorption rate of *CS4* molecules increased or became higher than their adsorption in a phenomenon called desorption potential. This resulted as increase the dissolution rate of *CS*-metal more than protection by inhibitor^16^.

**Table 3:** Tafel parameters for *CS*-metal before and after the addition of various concentrations of *CS4* compound at room temperature.

*Inh*	Conc, *M*	*E*_corr_, (mV) Vs. Ag/AgCl	i_corr_ (mA/cm^2^)	*β*_a_ (mV/dec)	*β*_c_ (mV/dec)	*r* (mm/year)	θ	$\upeta$
Blank	00.00	− 493.224	0.6949	136.83	− 163.45	8.1281	–	–
*CS4*	1 ppm	− 487.562	0.1571	105.52	− 153.56	1.8414	0.773	77.39
	5 ppm	− 482.905	0.1215	100.27	− 127.04	1.4186	0.825	82.51
	15 ppm	− 480.301	0.0914	146.25	− 124.32	1.0653	0.868	86.84
	25 ppm	− 487.513	0.0668	118.93	− 124.36	0.7814	0.903	90.38
	35 ppm	− 484.424	0.0519	137.22	− 125.57	0.6076	0.925	92.53
	50 ppm	− 492.517	0.0299	129.01	− 121.11	0.3544	0.956	95.69

i_corr_ decreased with *CS4* addition till reached 0.1571 mA/cm^2^ and 0.0299 mA/cm^2^ in presence of 1 ppm and 50 ppm respectively compared with uninhibited corrosion current 0.6949 mA/cm^2^. This indicated the adsorption process of *CS4* compound over *CS*-metal forming barrier protective layer against corrosive HCl^50^. From Table 3, the change in *E*_corr_ values were less than 85 mV implying that *CS4* inhibitor acted as hybrid inhibitor (mixed-type inhibitor)^51^. The I-V curves shape didn’t change after the addition of *CS4* concentrations, in addition to there was slightly change in *β*_a_ and *β*_c_ values which mean that the corrosion mechanism of *CS*-metal didn’t change^52^. η values increased with rising concentration till reached 95.69% due to the adsorption of *CS4* molecules decreased the contact between *CS*-metal and aggressive solution by blocking the anodic and cathodic site at *CS*-metal during the adsorption process of *CS4* molecules and decrease the corrosion rate of *CS*-metal^25,51^.

### Adsorption isotherm

The adsorption of *CS4* molecules at *CS*-metal/1 M HCl interface can be explained by the replacement of water molecules by organic molecules (*CS4*) as the following equation:

12 $${CS4}_{sol}+n {\mathrm{H}}_{2}{\mathrm{O}}_{ads}\leftrightarrow {CS4}_{ads}+\mathrm{n} {\mathrm{H}}_{2}{\mathrm{O}}_{sol}$$

where *CS4*_*sol*_ and *CS4*_*ads*_ are organic molecules (*CS4* compound) in the solution (liquid phase) and adsorbed on *CS*-metal (adsorption phase), respectively, and n is the number of replaced water molecules^37^.

**Figure 8:**
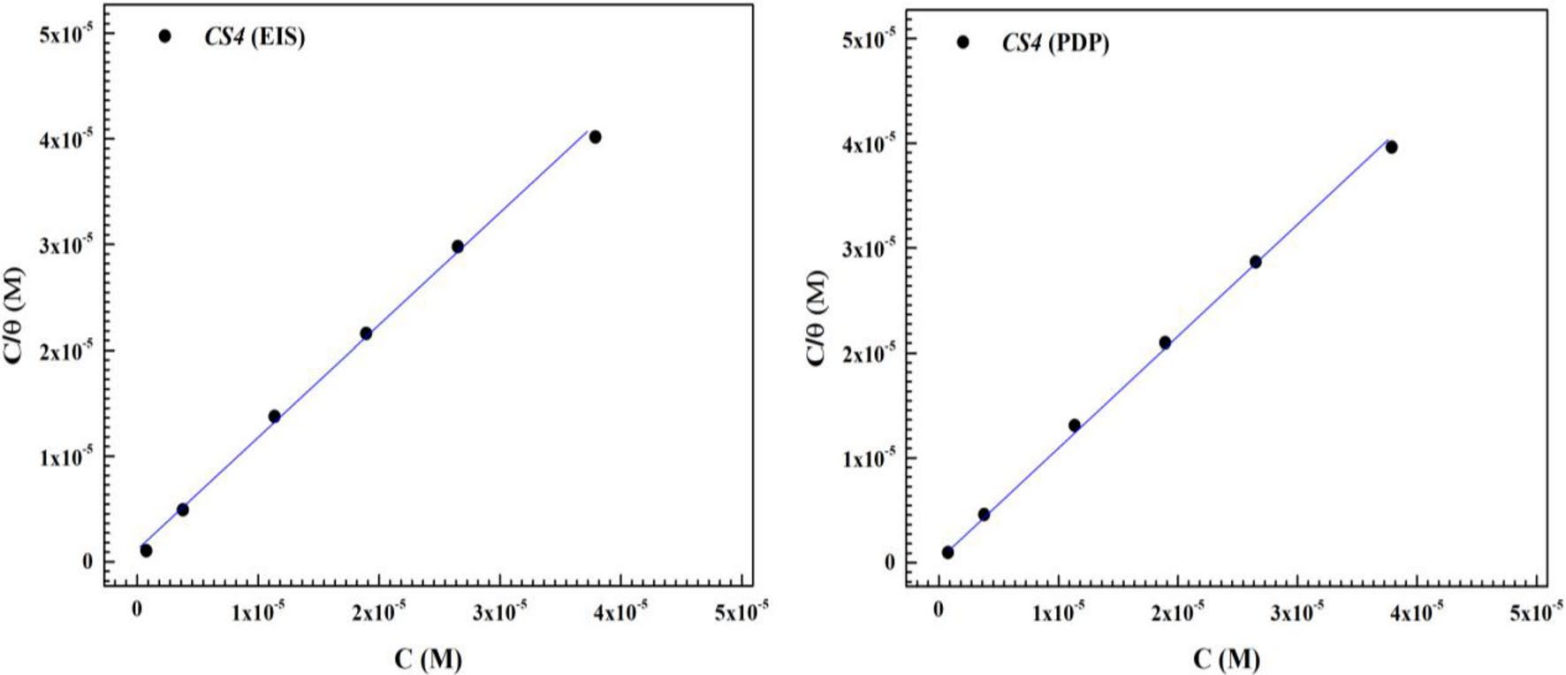
The linear form of Langmuir adsorption isotherm for *CS4* on *CS*-metal surface in 1 M HCl based on EIS and PDP data at room temperature.

The corrosion mitigation potency of organic compounds mainly depends on their adsorption capability over *CS*-metal surface. To clarify the adsorption properties (nature & strength) of *CS4* compound, the experimental data calculated from EIS and PDP were fitted to a various adsorption isotherms. Langmuir adsorption isotherm was the best fit isotherm with linear association coefficients very close to 1 (R^2^ = 0.9988) which was presented in Fig. 8 as a straight line with slope was nearly 1 (slope = 1.03). This indicated that, the adsorption of *CS4* compound on *CS*-metal surface obeys Langmuir adsorption isotherm. Also, a protective monolayer film of *CS4* formed over *CS*-metal and no interactions between adsorbed *CS4* molecules occurs^37,39^. The values of *K*_ads_ (adsorption equilibrium constant) and $\Delta {G}_{\mathrm{ads}}^{^\circ }$(standard free energy) were listed in Table 4 and were calculated from the following equations:

13 $$\mathrm{C}/\uptheta=\left(\frac{1}{{K}_{\mathrm{ads}}}\right)+\mathrm{C}$$

14 $$\Delta {G}_{\mathrm{ads}}^{^\circ }=-\mathrm{RTln}\left(55.5{K}_{\mathrm{ads}}\right),$$

where 55.5, *C*, $\uptheta ,$ R and T are molar concentration of water, the concentration of inhibitor, the surface coverage, the gas constant, the absolute temperature (*k*) and the standard free energy respectively. The high value of *K*_ads_, reflects the high adsorption ability of *CS4* compound and the high stability of the adsorbed layer on *CS*-metal^53^. $\Delta {G}_{\mathrm{ads}}^{^\circ }$ values that obtained from PDP and EIS data were − 44.071 kJ mol^−1^ and − 43.274 kJ mol^−1^ respectively, indicating a strong interaction between *CS4* and the surface of *CS*-metal. Also, the −ve sign reflected that the adsorption of *CS4* was spontaneous process^38^. It is known that, the value around − 40 kJ mol^-1^ or higher related to formation of a new bonds (coordinate bond) between inhibitor and *CS*-metal through charge transfer or sharing (chemisorption)^22^. $\Delta {G}_{\mathrm{ads}}^{^\circ }$ value indicating that, chemical adsorption of *CS4* over the surface of *CS*-metal via hetero atoms (N & O) and aromatic ring in its structure^30,33^.

**Table 4:** Langmuir adsorption isotherm parameters for *CS4* adsorption on *CS*-metal at room temperature.

Technique	Slope	R^2^	K, (L.mol^−1^)	$\Delta {G}_{\mathrm{ads}}^{^\circ }$(kJmol^−1^)
PDP	1.0408	0.9988	1296553	− 44.071
EIS	1.0564	0.9975	934827.5	− 43.274

### Computational studies

#### Frontier molecular orbitals

Quantum chemical calculations were used to understand how the material's structural and electrical characteristics affect the way it prevents corrosion. Additionally, the study’s objective was to understand more about the donor–acceptor interactions between inhibitor molecules and metal atoms. The frontier molecular orbitals and charge density distributions of chemical compounds can be used to help comprehend their molecular reactivity. The E_HOMO_ orbital is most highly associated to an inhibitor’s ability to give electrons, whereas the E_LUMO_ orbital is most typically associated to an inhibitor's capacity to receive electrons. High values of EHOMO are thought to represent a molecule’s propensity to give electrons to suitable acceptor molecules with energy level and an empty molecular orbital rather than a molecule’s propensity to strongly accept electrons from other molecules. Therefore, a molecule has a higher chance of receiving electrons the lower the ELUMO value is. The binding capacity of the inhibitor to the metal surface is increased by lowering the ELUMO energy values and raising the EHOMO energy values. As a result, the metal surface’s ability to accept charges rises, which causes a specific detail between the inhibitor’s acceptor anti-bonding orbital and the donor iron atoms.

**Figure 9:**
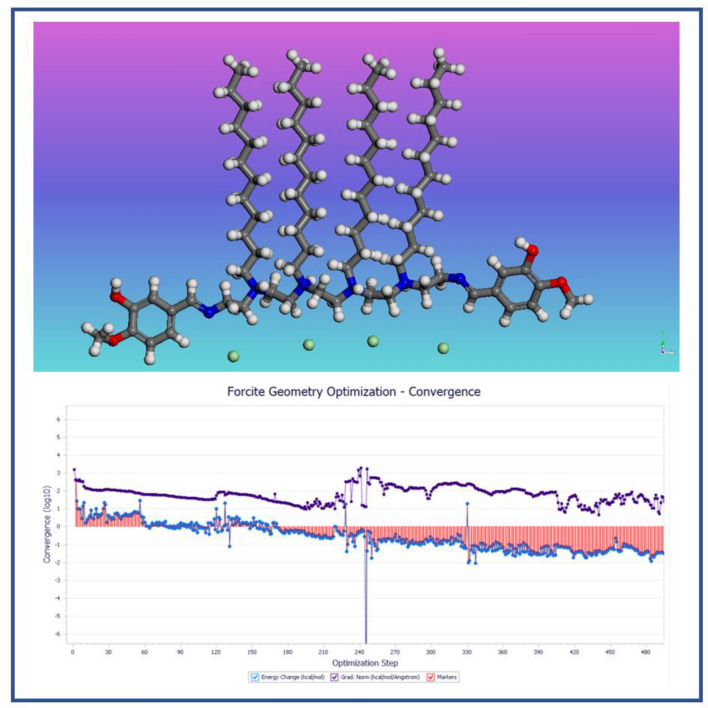
Optimized geometry of the investigated *CS4* compound.

HOMO and LUMO values explained the ability of *CS4* molecules to donate and accept electrons. The value of ΔE (energy gap) of FMO (Frontier Molecular Orbital) gives information about the kinetic stability and chemical reactivity of *CS4* structure. Also, FMO helps in the prediction of the most reactive sited in *CS4* structure. Figure 9 show the optimized geometry of the investigated compound. Some quantum chemical parameters of the studied *CS4* were calculated based on HOMO and LUMO values such as: I (ionization potential), A (electron affinity) x (electronegativity) and η_H_ (chemical hardness) and listed in Table 5.

**Table 5:** Quantum chemical calculated parameters of the investigated *CS4*.

Quantum parameters	(Gas phase)	(Liquid phase)
E_HOMO_ (eV)	− 7.01	− 7.45
E_LUMO_ (eV)	0.76	0.91
ΔE (eV) = ELUMO − EHOMO	7.77	8.36
µ (Debye)	5.43	7.83
I(eV) = − E_HOMO_	7.01	7.45
A(eV) = − E_LUMO_	− 0.76	− 0.91
X(eV) = − 1/2(E_HOMO_ + E_LUMO_)	3.12	3.27
η(eV) = − 1/2(E_HOMO_ − E_LUMO_)	3.88	4.18
σ = 1/η_H_ ≅ − 2/(E_HOMO_ − E_LUMO_)	0.257	0.239
ΔN = χ_Fe_ − χ_inh_/2(η_Fe_ − η_inh_)	0.768	0.795

**Figure 10:**
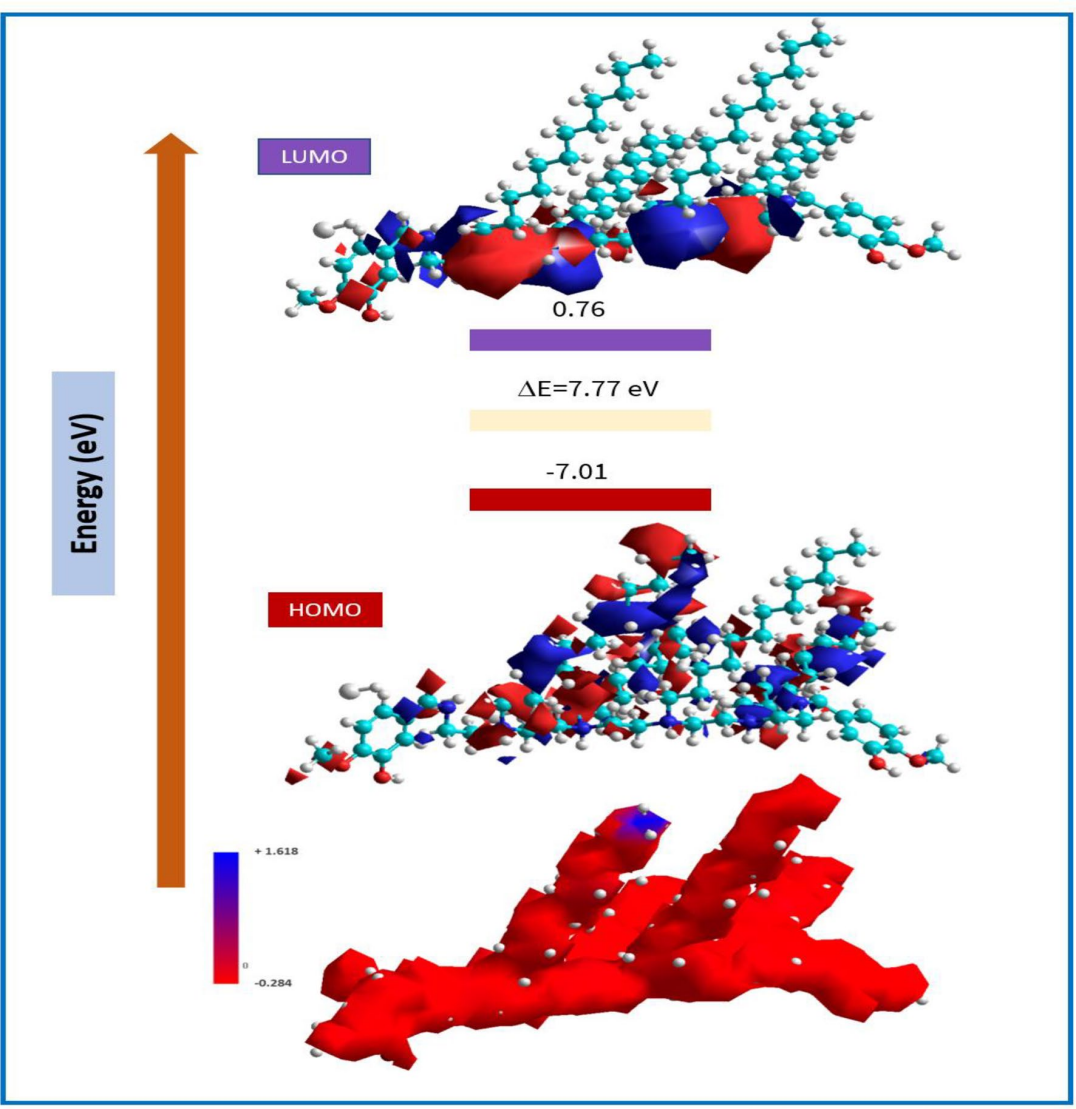
HOMO and LUMO surfaces as well as the electrostatic potential (ESP) for *CS4* compound.

The HOMO and LUMO densities were found in the Nitrogen-containing region of *CS4* structure as seen in Fig. 10 are responsible for the electron sharing (donation and acceptation) between *CS4* and Fe (1 1 0) surface^54–56^.

ΔE value explained the relationship of *CS4* electronic properties with chemical structure such as frontier electron density and chemical stability by explanation of charge transfer^57^. The high value of ΔE (7.77 eV) reflects the metal ion complex’s stability over the Fe surface. The graphical representation of molecular orbitals can provide insight into the aromaticity and lone pair. The + ve and − ve phase of wave-functions are represented in red and blue colour, respectively.

A significant correlation may be shown in terms of molecular hardness; that is, when molecular hardness decreases, molecules react with surfaces more easily and have a smaller amount of corrosion-causing power. The molecule with the largest dipole moment is also more effective in inhibiting other compounds. It was discovered that the quantum indices, such as the magnitudes of A and I, play an important role in evaluating the effectiveness of the *CS4*. The lower the values of (I), the greater the ability of the inhibitor molecule to offer electrons to the surface of *CS*-metal. In a similar fashion, having high values of (A) encourages the inhibitor to host the electrons that have been accepted from the substrate surface. The fact that the N value is 0.768 (< 3.6), indicates that the inhibition efficiency related to the ability of *CS4* to donate electrons^22^. Another index that is used to measure the likelihood of bond formation is the dipole moment, denoted by the symbol. There is a lot of controversy surrounding the application of µ values to correlate the experimental findings for the inhibition performance. The higher µ values, the lower the inhibition efficiency, which reflects that lower µ will help in accumulation of *CS4* molecules over *CS*-metal surface. On the other hand, the other view suggests that, the higher µ values related to a higher interaction between *CS4* molecules and *CS*-metal surface which will increase the inhibition performance. One of the contending opinions is that higher dipole moment values result in lower inhibition efficiency. The theoretical approach is consistent with the quantifiable parameters that have been discussed.

##### Molecular electrostatic potential (MEP)

MEP is a tool that was used for predicting the nucleophilic and the electrophilic attack sites, visualize the charge distribution and charge-related characteristics of target inhibitors A colour grading system was used to illustrate the molecular interactions and chemical composition. MEP surface analysis of *CS4* was determined using DFT calculations with the optimized structure and B3LYP/6-31G + (d, p) basis set. Fig. 10 illustrates the electrostatic potential surface of the investigated *CS4*. The compound’s colour code is in the range of 0.284e3 to +1.618e3. The MEP structure’s red and blue colours denote more electron-rich and electron-poor regions, respectively. The polarization effect was observed, the − ve potential areas of the MEP are localized around electronegative atoms (oxygen and nitrogen), while the + ve potential regions are localized around hydrogen atoms. The + ve electrostatic potential and the negative electronegative potential sites are more favourable for the attraction of nucleophilic and electrophilic species.

##### Monte Carlo (MC) and molecular dynamic (MD) simulation

**Figure 11:**
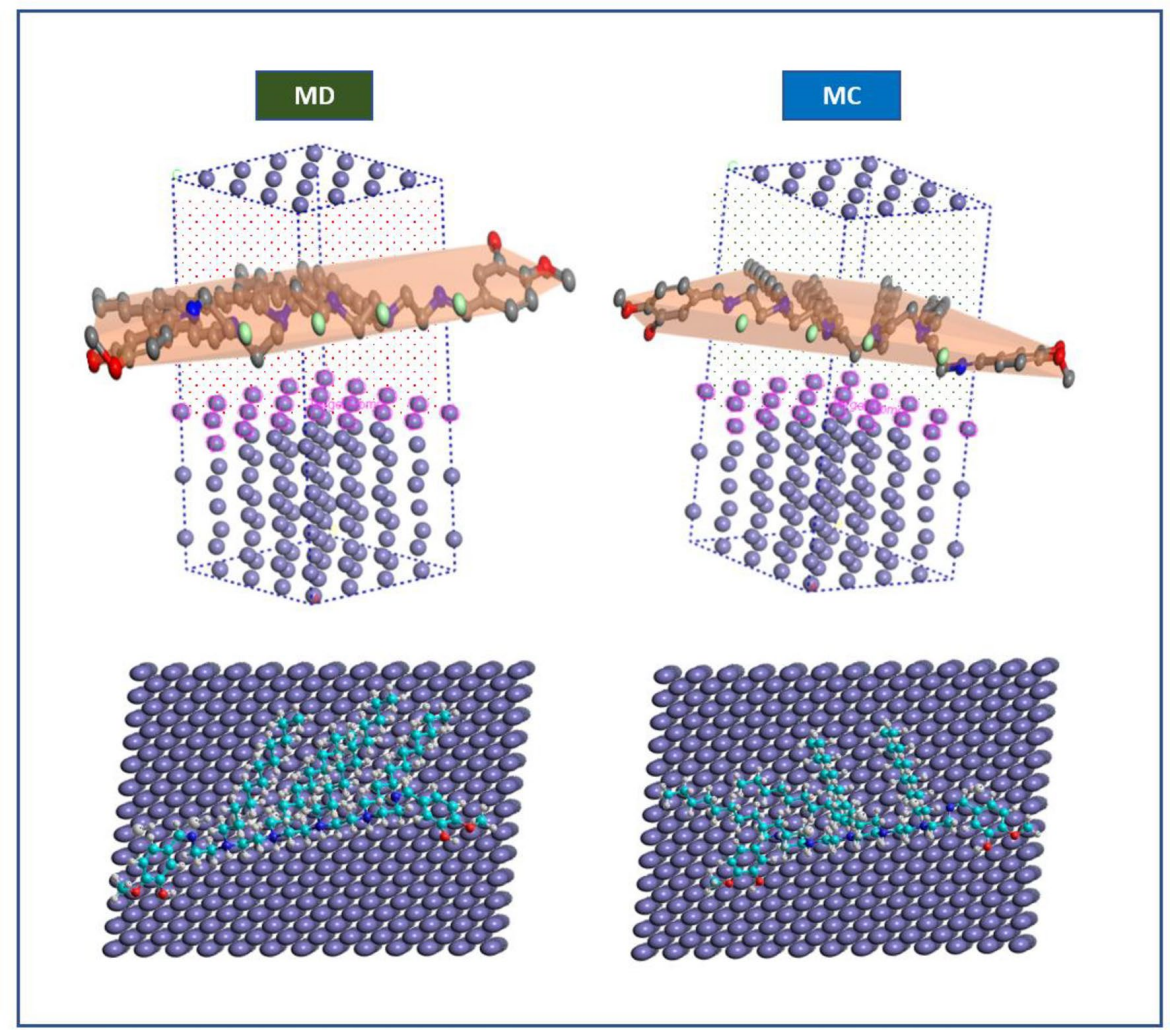
MD and MC obtained from the adsorption configurations of *CS4* on Fe (110) surface.

Using the simulated corrosion environment, the lowest energy configurations of the various protonated forms of *CS4* on *CS*-metal surface were determined as shown in Fig. 11. Based on the adsorption geometries, heteroatoms (O and N) were involved in the *CS4* adsorption process. *CS4* adsorption affinity resulted in the formation of a protective anticorrosion layer over *CS*-meal surface. The adsorption energies were calculated using the following Equation^57,58^:

15 $${E}_{ads}={E}_{Fe \left(110\right)inh}-({E}_{Fe \left(110\right)}+{E}_{inh})$$

where ${E}_{Fe \left(110\right)inh}{, E}_{Fe \left(110\right)}$ and ${E}_{inh}$ are the total energy of the simulated corrosion system, the total energy of Fe (1 1 0) surface and that *CS4* molecule, respectively. The obtained data from the simulation method reveals that:

- ${E}_{tot}$ (total energy) is equal to the summation of the internal and the adsorption energy of the adsorbate.
- ${E}_{ads}$ (adsorption energy) is the energy released when the adsorbate (*CS4* molecule) is relaxed on the substrate (*CS*-metal surface) and the equal rigid adsorption energy plus the deformation energy.
- ${E}_{rig}$ (rigid adsorption energy) is the energy released (or required) when the unrelaxed adsorbate *CS4* molecules are adsorbed on the substrate.
- ${E}_{def}$ (deformation energy) is the energy released when the adsorbed *CS4* molecules are relaxed on the *CS*-metal surface.

The adsorption energy of *CS4* was listed in Table 6, indicating a more stable and stronger interaction between the Fe surface and *CS4*, and therefore better inhibition efficiency.

**Table 6:** The output energies calculated by Monte Carlo simulation for *CS4* on Fe (110).

*Inh*	$E_{tot} \,\left( {{\text{kJ mol}}^{{ - {1}}} } \right)$	$E_{ads} \,\left( {{\text{kJ mol}}^{{ - {1}}} } \right)$	$E_{rig} \left( {{\text{kJ mol}}^{{ - {1}}} } \right)$	$E_{def} \left( {{\text{kJ mol}}^{{ - {1}}} } \right)$	$\left( {{\text{d}}E_{{{\text{ads}}}} /{\text{dNi}}} \right)$
*CS4*	− 170.3115	− 133.09	− 591.186	− 739.722	1.33.09

**Figure 12:**
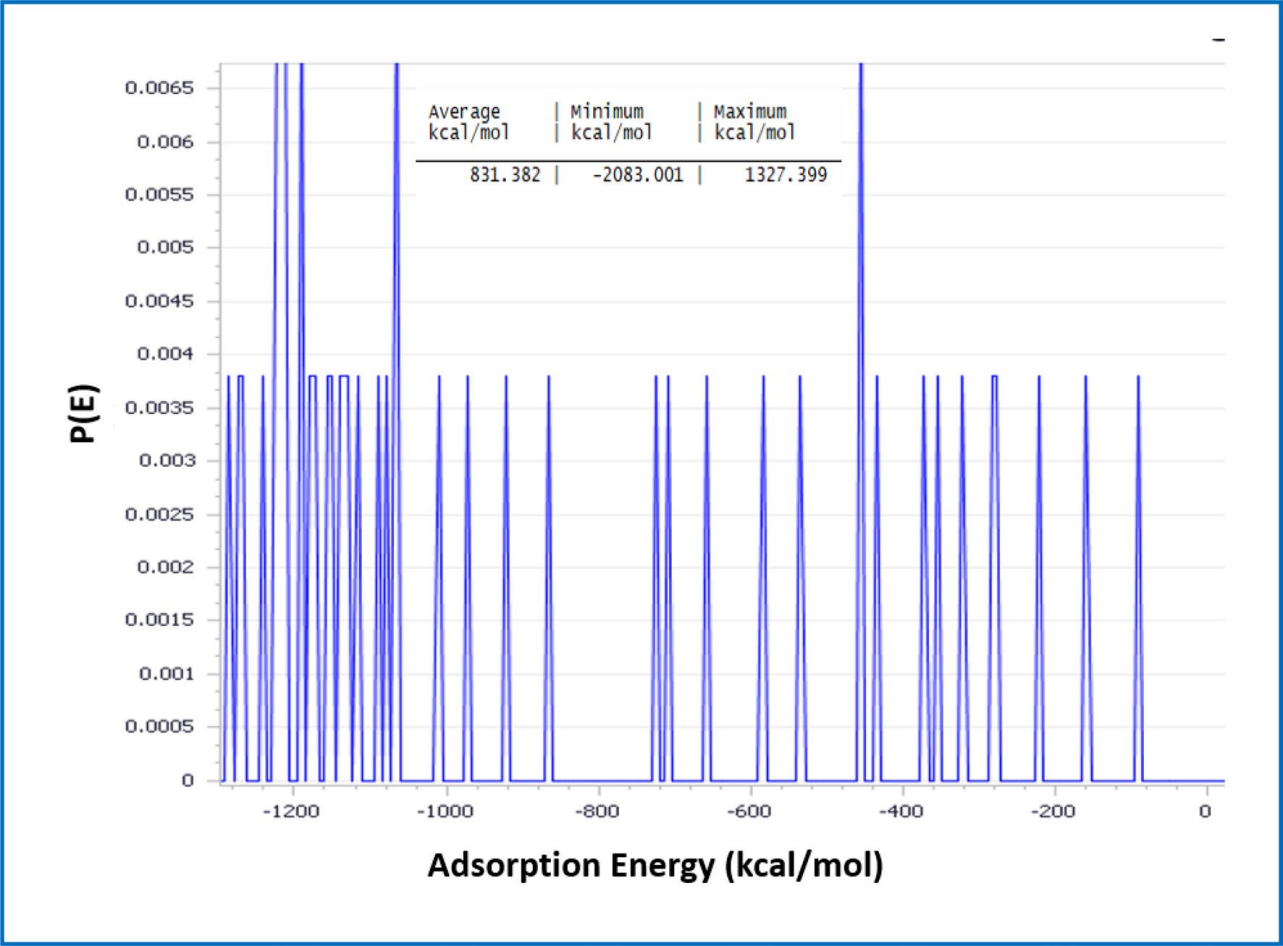
Distribution of the adsorption energies of *CS4* on to *CS*-metal surface obtained via MC.

The theoretical data obtained from Monte Carlo simulations were matched with the experimental data. The –ve value of the adsorption energies as seen in Fig. 12, indicated that *CS4* molecules adsorbed over *CS*-metal surface spontaneously^59,60^.

To examine the interaction between *CS4* and metal surfaces, MD simulations were used to predict the binding energy ${({\varvec{E}}}_{{\varvec{b}}{\varvec{i}}{\varvec{n}}}$) of *CS4* on the Fe surface and explains the significant association between experimental inhibition efficiency and binding energy the studied *CS4*. The interaction energy (${E}_{int}$) between the *CS4* molecules and Fe (110) surface has been obtained^61,62^.

The calculated ${{\varvec{E}}}_{{\varvec{b}}{\varvec{i}}{\varvec{n}}}$: ${E}_{ads}={-E}_{inh}=-{E}_{ads}$ was listed in Table 7, indicated that a strong interaction between *CS4* and *CS*-metal surface occurred and more stable inhibitor/surface interaction.

**Table 7:** Binding energies of the studied *CS4* adsorbed on Fe (110) surface.

*Inh*	$E_{bin} \,\left( {{\text{kJ}}\,{\text{mole}}^{{ - {1}}} } \right)$
*CS4*	2034.42

### Surface analysis (SEM)

**Figure 13:**
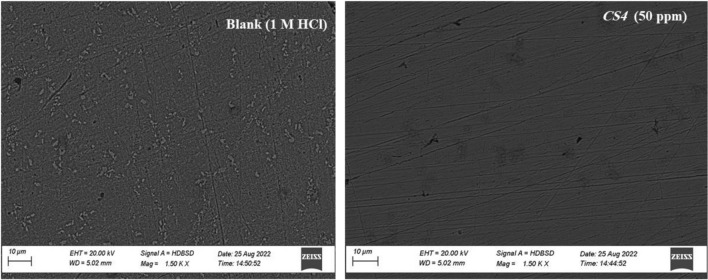
SEM images of the uninhibited (1 M HCl) and the inhibited (50 ppm *CS4*) *CS*-metal after 6 h at room temperature.

The surface morphology examination of the *CS*-metal surface in 1 M HCl solution with and without the optimum concentration (50 ppm) of *CS4* compound was performed by *SEM* as in Fig. 13. The blank solution (free acid) showed the badly damaged surface due to the aggressive action of corrosive media (1 M HCl) as result in dissolution of *CS*-metal (corrosion process) and roughness surface layer was observed. In contrast, the appearance of *CS*-metal surface was different in presence of *CS4* compound. It can be seen that after the addition of *CS4* compound, an improvement in carbon steel surface morphology (more smooth) and less damaged and roughness surface was observed, resulting in good inhibition performance of *CS4* which reduce the corrosive effect of HCl. This may be due to the adsorption process over *CS*-metal forming a protective barrier layer which decreases the contact between *CS*-metal and aggressive solution^37,38,43^.

### XPS analysis

**Figure 14:**
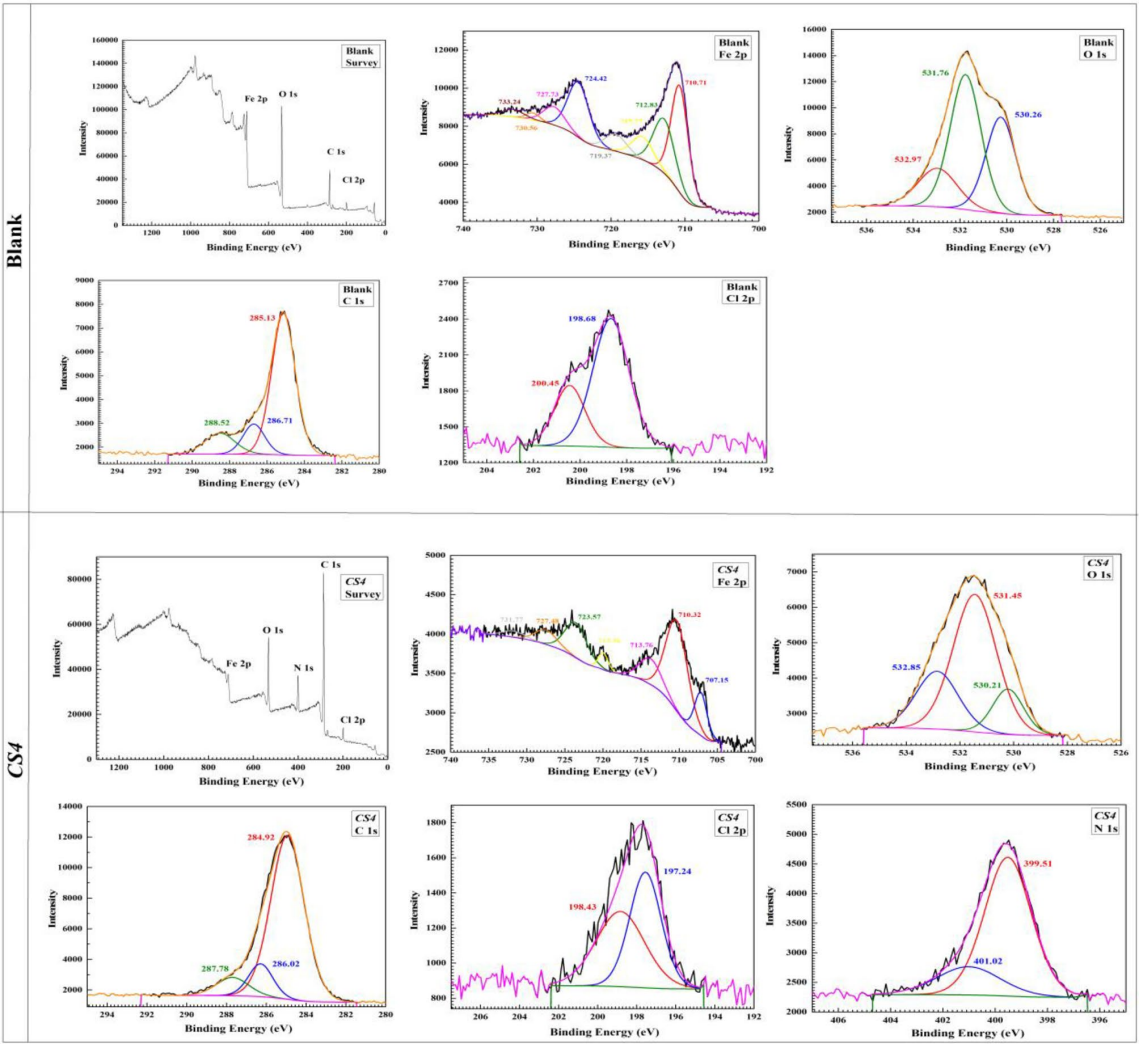
XPS analysis for *CS*-metal surface in absence and presence of *CS4*.

XPS measurements were performed on both inhibited and uninhibited *CS*-metal as shown in Fig. 14. The existence of Fe, O, and Cl elements in the XPS results of *CS*-metal samples in blank solution (without *CS4*) reflected the occurrence of *CS*-metal dissolution (corrosion process) in 1 M HCl. Figure 14 presented the spectrum of Fe 2p revealed satellite peaks at 730.56 and 719.37 eV. The peaks at 710.71, 712.83, 715.77, 724.42, and 727.73 eV corresponded to Fe_2_O_3_, FeOOH, FeCl_3_, FeO, and Fe_3_O_4_, respectively^63,64^. The XPS spectra of *CS*-metal treated with *CS4* inhibitors showed Fe at 707.15 eV indicating that the high surface coverage provided by *CS4* molecules which can effectively isolate Fe atoms from corrosive HCl solution^65^.

As in Figs.14, the spectrum of O 1 s revealed satellite peaks at 530.26 and 531.76 eV, which could be attributed to the corrosion products such as iron oxides (Fe_2_O_3_ and Fe_3_O_4_) and hydrous iron oxides (FeOOH) respectively. The presence of O–Fe bond indicating the occurrence of donor acceptor interactions between the vacant d orbitals of Fe and sp^2^ electron pairs of OH and O-CH_3_ group. The peak at 532.97 eV could be attributed to oxygen of adsorbed H_2_O and C–O bond. Also, the intensity decreased gradually, thereby indicating that addition of *CS4* reduces the erosion of corrosion particles to some extent^65,66^.

Figure 14 represents C1s spectrum of *CS*-metal surface in presence of *CS4* molecules at 284.92, 286.02 and 287.78 eV. The peak at 284.92 eV could be attributed to C–C, C = C and C–H bonds, the peak at 286.02 eV can be attributed to C–O bond and the peak at 287.78 eV can be partly attributed to aromatic rings (π-π * shakeup satellite) and partly associated to the coordination of oxygen with Fe of *CS*-metal surface. These bonds exist in *CS4* compound, proving that *CS4* compound adsorbed and shielded *CS*-metal surface from corrosive HCl solution.

Cl 2p spectrum as in Fig. 14 has included two peaks at 197.24 eV and 198.43 eV associated to Cl–Fe bond in FeCl_3_^67^.

N 1 s spectrum as in Fig. 14 revealed satellite peaks at peaks at 401.02 and 399.5 eV, which could be attributed to N–Fe and N–C, respectively^54,55^. This result indicated that, the lone electron pairs of distributed N atoms in *CS4* inhibitor formed chemical bonds with the empty Fe d-orbital which allow *CS4* molecules to adsorb onto the *CS*-metal surface^65^.

### The inhibition mechanism

It is known that, the organic inhibitors have suppression performance due to their adsorption at the metal/solution interface via their chemical structure, the charge distribution of the inhibitor, nature, and charged metal surface. Generally, the single adsorption mechanism between the inhibitor (*CS4*) and carbon steel surface is unachievable because of the complexity of the studied inhibitor’s structure. Therefore, the adsorption mechanism of *CS4* can be explained in 3 modes of adsorption:

1. Physical adsorption: electrostatic interaction which helps *CS4* molecules to be adsorbed on metal surface between + ve quaternary N (cationic head) of *CS4* and − ve sites of carbon steel surface (cathodic sites → cathodic inhibition) besides electrostatic interaction between chloride ions Cl^-^ and + ve sites of carbon steel (anodic sites → anodic inhibition). *CS4* has a high inhibition efficiency due to having many charged parts in its structure.
2. Chemical adsorption: formation of co-ordination bond between hetero atoms (N and O) and π-electrons (C = N and pyridine ring) with vacant d-orbital of carbon steel through electron sharing process (donor–acceptor interactions).
3. Aliphatic chain: the hydrophobic chains play an important role in the mitigation process, not only increase the thickness of the adsorption film layer but also enhances its density. *CS*-metal was inhibited effectively by *CS4* due to the presence of multiple numbers of hydrophobic chains in its structure. The aliphatic chains attached to quaternary N^+^ forces molecule towards carbon steel surface by displacement of water molecules and increase the surface coverage of *CS*-metal away from the corrosive effect of HCl solution and forms more compacted and denser protective layer.

## Conclusion

A newly tetra-cationic surfactant (*CS4*) was synthesized through two simple steps by condensation reaction of vanillin with pentaethylenehexamine followed by quaternization reaction of chlorododecane with the obtained Schiff-base in molar ratio 1:4. The main objective of the present work was to study the corrosion performance of the prepared *CS4* for carbon steel in 1 M HCl. *CS4* exhibited high inhibition efficiency was 95.69% which can be explained by the highly adsorption capability of *CS4* molecules over *CS*-metal surface. The adsorption of *CS4* followed Langmuir adsorption isotherm which can explained the chemical adsorption of *CS4* over *CS*-metal via hetero atoms in N = C, OH, NH and O-CH_3_ besides presence of the aromatic rings which can be explained by the high *K*_ads_ and $\Delta {G}_{\mathrm{ads}}^{^\circ }$ values. Various electrochemical techniques such as EIS and PDP that were carried out were matched with other which suggested that, *CS4* acted as mixed type inhibitor according to Tafel data. Also, the theoretical quantum studies (DFT, MC and MD) confirmed the adsorption capacity of *CS4* over Fe (110) in 1 M HCl as well as SEM and XPS analysis.

## Data Availability

All data generated or analysed during this study are included in this manuscript.
