# Author Correction: Theoretical and electrochemical evaluation of tetra-cationic surfactant as corrosion inhibitor for carbon steel in 1 M HCl

**DOI:** 10.1038/s41598-023-33409-3

**Published:** 2023-04-19

**Authors:** A. Elaraby, Shrouk. Abd El-samad, Eman. A. khamis, E. G. Zaki

**Affiliations:** 1 Egyptian Petroleum Research Institute, Nasr City 11727, Cairo, Egypt; 2 University College of Umluj, University of Tabuk, Tabuk, Saudi Arabia

Correction to: *Scientific Reports* 10.1038/s41598-023-27513-7, published online 18 January 2023

The original version of this Article contained an error in Affiliation 2, which was incorrectly given as ‘University College of Umluj-Tabuk University, Umluj, Saudi Arabia’. The correct affiliation is listed below:

University College of Umluj, University of Tabuk, Tabuk, Saudi Arabia.

The original Article has been corrected.

